# Exploring Patient Multimorbidity and Complexity Using Health Insurance Claims Data: A Cluster Analysis Approach

**DOI:** 10.2196/34274

**Published:** 2022-04-04

**Authors:** Anna Nicolet, Dan Assouline, Marie-Annick Le Pogam, Clémence Perraudin, Christophe Bagnoud, Joël Wagner, Joachim Marti, Isabelle Peytremann-Bridevaux

**Affiliations:** 1 Center for Primary Care and Public Health (Unisanté) University of Lausanne Lausanne Switzerland; 2 Groupe Mutuel Martigny Switzerland; 3 Department of Actuarial Science Faculty of Business and Economics, and Swiss Finance Institute University of Lausanne Lausanne Switzerland

**Keywords:** multimorbidity, pharmacy cost groups, cluster analysis, claims data, patient complexity, health claims, informatics

## Abstract

**Background:**

Although the trend of progressing morbidity is widely recognized, there are numerous challenges when studying multimorbidity and patient complexity. For multimorbid or complex patients, prone to fragmented care and high health care use, novel estimation approaches need to be developed.

**Objective:**

This study aims to investigate the patient multimorbidity and complexity of Swiss residents aged ≥50 years using clustering methodology in claims data.

**Methods:**

We adopted a clustering methodology based on random forests and used 34 pharmacy-based cost groups as the only input feature for the procedure. To detect clusters, we applied hierarchical density-based spatial clustering of applications with noise. The reasonable hyperparameters were chosen based on various metrics embedded in the algorithms (out-of-bag misclassification error, normalized stress, and cluster persistence) and the clinical relevance of the obtained clusters.

**Results:**

Based on cluster analysis output for 18,732 individuals, we identified an outlier group and 7 clusters: individuals without diseases, patients with only hypertension-related diseases, patients with only mental diseases, complex high-cost high-need patients, slightly complex patients with inexpensive low-severity pharmacy-based cost groups, patients with 1 costly disease, and older high-risk patients.

**Conclusions:**

Our study demonstrated that cluster analysis based on pharmacy-based cost group information from claims-based data is feasible and highlights clinically relevant clusters. Such an approach allows expanding the understanding of multimorbidity beyond simple disease counts and can identify the population profiles with increased health care use and costs. This study may foster the development of integrated and coordinated care, which is high on the agenda in policy making, care planning, and delivery.

## Introduction

Health care systems worldwide are facing considerable challenges from the increasing number of chronic and multimorbid patients, characterized by complex needs and frequent transitions between care settings [1]. In Switzerland, 2.2 million people report a chronic disease and nearly 20% of the population older than 50 years have multiple chronic diseases (multimorbidity) [2]. Although the trend of progressing multimorbidity is widely recognized [3-6], it is still unclear how best to take care of patients with multimorbidity and which interventions would be effective. For more than two decades, integrated and coordinated care have been developed worldwide [7]. Nevertheless, integrated and coordinated care faces continuing challenges such as scaling-up, implementation, and sustainability difficulties. Additionally, integrated and coordinated care requires development of novel approaches to evaluate and measure patients multimorbidity and complexity. This is key to stratify the targeted population and adapt the intervention to the needs of the patients. Often, such evaluations and measures rely on morbidity indices (eg, Charlson and Elixhauser) or on the number of (self-reported) chronic conditions or comorbidities [8]. Whereas the former were developed in an inpatient setting as predictors of mortality, the latter may not comprehensively reflect the patient’s disease burden and complexity. Despite these limitations, they remain often used because of their relative accessibility and simplicity. In settings where electronic medical (health) records, national disease registries, or data on chronic conditions are unavailable, administrative health insurance claims data represent a potentially useful source of information. In fact, they are increasingly used in health services research, especially to express multimorbidity using pharmacy-based cost groups (PCGs) [9,10]. PCGs, based on use of prescribed drugs rather than on clinical information, were developed as a proxy for morbidity measure [11]. Although the approach has limitations related to underestimation of medicines used, unclaimed, or paid out-of-pocket and thus not present in the data or the assumption that the drug is used exclusively for treating the particular condition [11,12], it allows mapping patient profiles to reflect their morbidity status. As such mapping approaches and comorbidity counts are considered simplistic [13], researchers may consider alternative methods to investigate patient complexity more exhaustively. One such method is cluster analysis, which relies on the idea that many common conditions cluster together in the population in predictable patterns [13]. It has been shown that cluster analysis of real-world data for drug use research can be used for detecting clinically plausible subgroups [14]. Similar approaches of classifications based on multimorbidity patterns have been applied in the literature [14-16], but using PCGs as the multimorbidity indicator for cluster analysis is novel. In that context, the aim of our study is to investigate patient multimorbidity and complexity beyond simple mapping and counts of PCGs, using clustering methodology in claims data of Swiss residents aged ≥50 years.

## Methods

### Data Source and Sample

We included data of 240,511 insured people aged ≥50 years continuously enrolled in one of the largest health insurance companies in Switzerland, Groupe Mutuel, for the 2015-2018 period. In addition to demographic information (age and gender), data contained PCGs for each individual, costs covered by the patient (cost sharing), type of health insurance model (with or without gatekeeping), and reimbursed health care services: number of visits to various physicians with associated costs and physicians’ specialization and hospitalizations. To identify insured persons with cost-intensive, chronic diseases and correspondingly high health care use based on their drug consumption, health insurance companies are translating the drug use data reflecting active ingredient and quantity, based on Anatomical Therapeutic Chemical and defined daily dose, into the PCGs. This procedure was developed and officially accepted by the Federal Office of Public Health in Switzerland [17]. In our study, the patients were classified as multimorbid when they were assigned two or more PCGs, based on their yearly drug use.

### Ethical Considerations

Data were deidentified by the insurance company to guarantee anonymization, and ethical approval for this study was waived by the Cantonal Commission for the Ethics of Research on Human Beings (Lausanne, Switzerland).

### Cluster Analysis

We adopted a clustering methodology based on random forests (RFs) [18]—a popular classification and regression tree-based method—that includes several steps and machine learning algorithms [19-21]. The methodology is inspired by a clustering methodology designed by Breiman and Cutler [19], the creators of RFs [20,21].

In a preprocessing step, we extracted 34 PCGs as the only input feature for the clustering procedure. We grouped the 34 PCGs into 15 disease categories, which were valued meaningful from a clinical perspective (Multimedia Appendix 1). We then considered the first year of information only, and extracted a 10% random sample, to allow for effective processing for the computationally expensive steps. To confirm the results, the random sampling was performed multiple times, which led to similar clusters. Finally, we discarded points showing no PCG or only one type of PCG. Since we ultimately use an algorithm to detect clusters based on density given by the distances between points, the presence of many identical points at the same positions may perturb the algorithm and unnecessarily make the computation more expensive. Keeping a small random sample of these points would reduce the perturbation but not change the results while adding a dispensable complication, notably for the hyperparameter selection needed to detect these additional clusters.

To initiate the clustering procedure, we created a synthetic data set of the same size as the original data, by random sampling from the distributions of each input variable within the data. The idea is then to train an RF model to classify synthetic and original points, with the aim of taking advantage of the *proximity* measure, an embedded RF metric of similarity between points. An RF aggregates the prediction of multiple decision trees (DTs) by considering the class they predict in majority. DTs are classification models that separate the data points into subspaces (leaves) by imposing thresholds on the input variables and predicting the class within each subspace as the majority class. The proximity between two points is then computed as the number of times they fall in the same leaf across the trees in the forest. To stabilize the random effects of RFs, we trained 10 RF models, computed the proximities for all pairs of points for each model, and averaged them to obtain a mean proximity matrix characterizing the data. We then used multidimensional scaling (MDS) [22] to project the corresponding distance matrix (1 – proximity matrix / (number of trees)) in 2D while preserving the distances and allow for visualization of the resulting clusters. Finally, we applied hierarchical density-based spatial clustering of applications with noise (HDBSCAN) [23] to detect clusters within the obtained 2D data, after discarding the synthetic points from the data. HDBSCAN extracts clusters as dense gatherings of points separated by sparse regions with few points. Given that no cross-validation is possible with clustering methodologies, reasonable hyperparameters were chosen for the RF, MDS, and HDBSCAN steps based on various metrics embedded in the algorithms and the clinical relevance of the obtained clusters. The metrics includes the *out-of-bag* (*OOB*) misclassification error, which shows how well RF differentiates the original data from the synthetic one. The outcome reflects how much structure there is in the data [19]. Another metric was *normalized stress*, measuring whether the distances between points are reasonably preserved after projection [22], and the *cluster persistence*, HDBSCAN embedded metrics indicating how well the clusters are defined and separated from each other [23]. In practice, we used the HDBSCAN and Scikit-learn libraries (in Python) for the final clustering and all previous steps.

## Results

**Figure 1:**
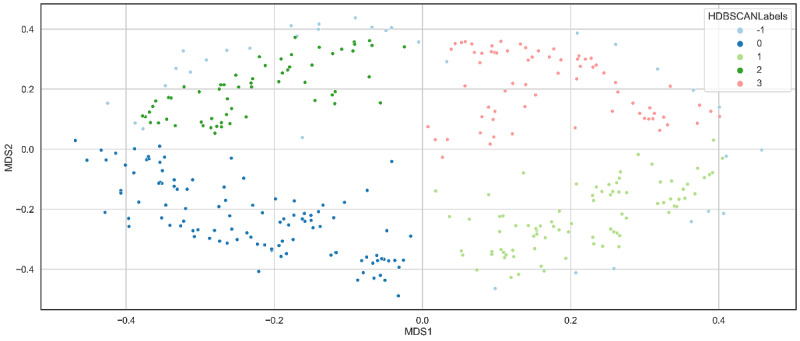
MDS projection of the data in two dimensions. The four clusters found by HDBSCAN are marked by the different colors and coded with the labels 0, 1, 2, and 3. The code –1 refers to the outliers. HDBSCAN: hierarchical density-based spatial clustering of applications with noise; MDS: multidimensional scaling.

**Figure 2:**
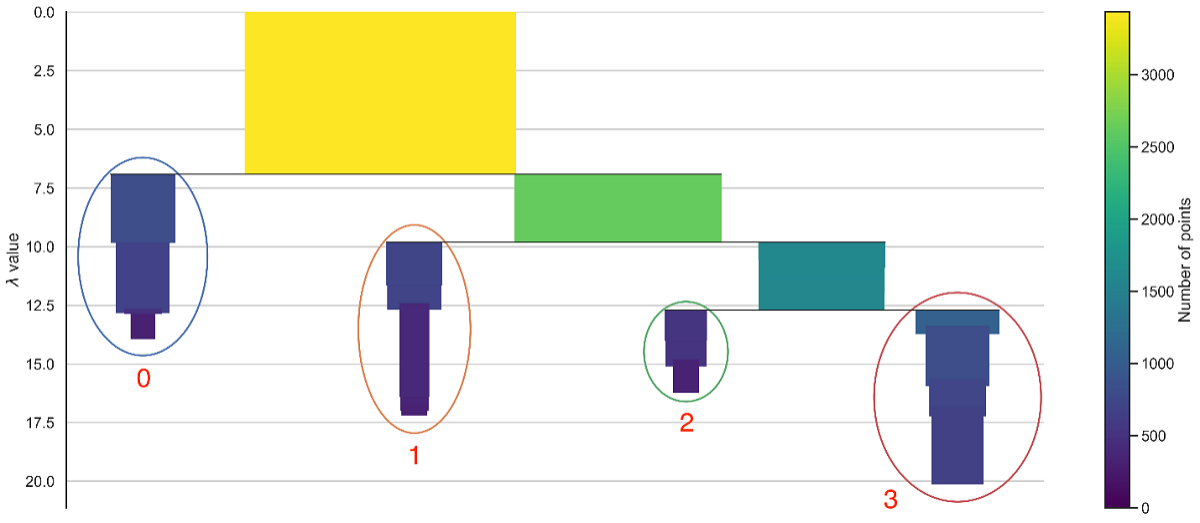
Condensed tree resulting from the hierarchical density-based spatial clustering of applications with noise algorithm performed on the data. Note: similar to a classical dendogram in a hierarchical clustering setting, the first yellow rectangle represents the entire data, which is split into two parts (called “branches”) when we reduce the maximum distance allowed between points within each branch (λ value = 1 / distance). Each rectangle represents a subpart of the data after a split and with a size proportional to the number of data points in the subpart. The entire data splits into cluster 0 and the green rectangle, which further splits into cluster 1 and a turquoise rectangle, when we reduce the distance allowed. The 4 detected clusters (signified by a circle and their number) are the branches that persist the most (do not split further, according to various rules of the algorithm) when the imposed maximum distance between points decreases while keeping a minimum size. The persistence is proportional to the length of the rectangles across the vertical axis. The tree can be interpreted as a probability distribution function upside down, with each cluster being a peak in the distribution.

After discarding individuals with missing information, our data set comprised 18,732 individuals (*points*). An initial examination of the data set exhibited three large “single” clusters that we extracted prior to the clustering procedure, showing no PCGs, only hypertension PCGs, and only mental disease PCGs, representing 67.9% (n=12,720), 9.7% (n=1813), and 4.1% (n=765) of the population, respectively. Clustering analyses, performed on the remaining 3434 patients not included in the latter “single” clusters, identified four distinct clusters: Cluster 0 to Cluster 3, numbered in the order in which they are detected while applying HDBSCAN (Figure 1). The clusters can be clearly visualized from this tree (Figure 2); and a good persistence of 0.29, 0.24, 0.15, and 0.24, respectively, was found. The average OOB misclassification error from the 10 RFs was 0.51, which is quite high, showing that RF does not differentiate well between the original and the synthetic data, and there is not much structure in the data. Regarding the performed MDS, the normalized stress was 0.31, indicating reasonable preserving of the distances between points.

**Figure 3:**
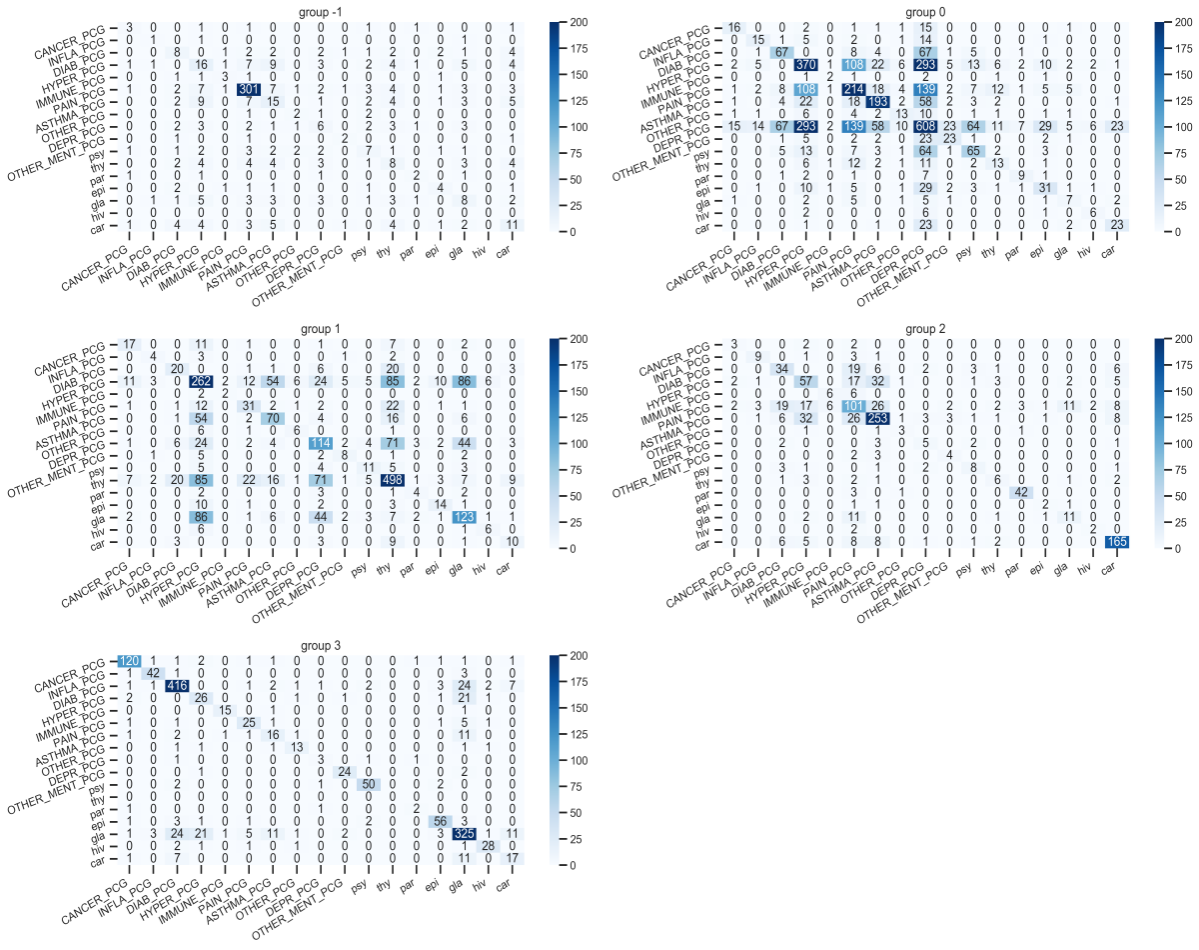
Joint distributions of PCGs within the 4 clusters (group 0-3) and outliers (group –1). PCG: pharmacy-based cost group.

The 4 detected clusters encompass different mixes of PCGs (Table 1 and Figure 3): Cluster 0 comprises a large mix of PCGs (mental + hypertension + pain + asthma [chronic obstructive pulmonary disease]) often appearing jointly; Cluster 1 comprises PCGs (thyroid, hypertension, glaucoma, and mix of others) appearing jointly less often; Cluster 2 comprises asthma, Parkinson, cardiac diseases, and pain rarely appearing jointly; and Cluster 3 comprises a large mix of PCGs almost never appearing jointly (single diseases).

The following description and interpretation of clusters is based on the descriptive statistics of health care use and costs data (Table 1), which help to understand the underlying principle of grouping individuals into PCG clusters. First, the members of Cluster 0 (n=817, 4.4%) had the highest number of PCGs and highest costs and health care use, and were referred to as “complex high-cost high-need patients” (for a detailed description, see Table 1). The degree of complexity in these settings was reflected as the combination of the following characteristics interpreted from descriptive statistics (Table 1): average number of PCGs, percentage of multimorbid patients, levels of health care use (eg, number of doctor consultations and hospital stays), and costs in the population subgroup. The members of Cluster 1 (n=709, 3.8%), although having multiple PCGs, had health care costs and use lower than in Cluster 0; thus, they were referred to as “slightly complex with inexpensive low-severity PCGs.” The members of Cluster 2 (n=531, 2.8%) were of the oldest age and presented especially high use of hospitalizations and visits to the generalist doctor and, thus, were referred to as “oldest at high risk.” High risk, interpreted in these settings from the descriptive statistics, was reflected by relatively high use of hospital care, yet lower than in the most complex cluster: long length of stay (5.6 and 6.6 nights for clusters “Oldest at risk” and “Complex high-cost high-need,” respectively) and high inpatient costs (CHF 2749 [US $2950] and CHF 3109 [US $3333], respectively). The members of Cluster 3 (n=1056, 5.6%) were characterized by a relatively small number of PCGs (close to 1) and the highest costs of medications and, thus, were referred to as “patients with 1 costly disease.”

**Table 1:** Descriptive statistics of clusters.

Statistics			All data		Outliers		Cluster 0 “Complex high-cost high-need”		Cluster 1 “Slightly complex with inexpensive low-severity PCGs^a^”		Cluster 2 “Oldest at high risk”		Cluster 3 “Patients with 1 costly disease”		No PCGs		Hypertension “Only hypertension”		Mental health “Only mental diseases”
Patients, n (%)			18,732 (100.0)		321 (1.7)		817 (4.4)		709 (3.8)		531 (2.8)		1056 (5.6)		12,720 (67.9)		1813 (9.7)		765 (4.1)
Age (years), mean (SD)			65.0 (10.6)		66.3 (10.8)		66.3 (10.6)		67.8 (10.2)		69.4 (10.9)		68.1 (11.2)		64.0 (10.4)		67.6 (9.7)		63.2 (10.9)
**Sex, n (%)**																			
	Men	8626 (46)		130 (40)		325 (40)		205 (29)		279 (53)		536 (51)		5772 (45)		1158 (64)		221 (29)	
	Women	10,106 (54)		191 (60)		492 (60)		504 (71)		252 (47)		520 (49)		6948 (55)		655 (36)		544 (71)	
Deductible (CHF; US $), mean			794 (852)		511 (548)		448 (481)		535 (574)		524 (562)		562 (603)		908 (974)		612 (657)		558 (599)
Model with gatekeeper^b^			0.5		0.4		0.4		0.4		0.4		0.4		0.5		0.5		0.5
Number of PCGs, mean			0.4		1.2		2.1		1.7		1.3		1.1		0.0		1.0		1.0
Multimorbid (yes)^b^			0.1		0.1		0.8		0.6		0.3		0.1		0.0		0.0		0.0
Ambulatory costs (CHF; US $), mean			5395 (5789)		7967 (8549)		11,731 (12,589)		7477 (8024)		9728 (10,439)		10,362 (11,120)		4074 (4372)		5462 (5861)		7571 (8125)
Inpatient costs (CHF; US $), mean			1419 (1523)		2134 (2290)		3109 (3336)		1811 (1943)		2749 (2950)		1575 (1690)		1199 (1287)		1372 (1472)		1585 (1701)
Costs of medications (CHF; US $), mean			1563 (1677)		2683 (2879)		4073 (4371)		2221 (2383)		3587 (3849)		4450 (4775)		965 (1036)		1732 (1859)		1961 (2104)
Total cost (CHF; US $), mean			8929 (9582)		13,684 (14,684)		19,950 (21,409)		12,440 (13,349)		17,057 (18,304)		17,312 (18,578)		6611 (7094)		9439 (10,129)		12,025 (12,904)
Number of days in the hospital, mean			2.6		4.3		6.6		3.6		5.6		3.4		2.0		2.4		3.5
Number of hospitalizations in a year, mean			0.2		0.4		0.5		0.3		0.4		0.3		0.2		0.3		0.3
Total number of consultations, mean			11.9		16.0		20.2		17.0		17.5		16.1		9.9		12.7		18.5
Number of consultations with generalist, mean			7.2		10.0		11.6		9.8		11.3		9.4		6.0		8.3		9.5
PCG groups in the cluster			All 34 PCGs		Mostly Pain		Mental + hypertension + pain + asthma (COPD^c^)		Thyroid + hypertension + glaucoma + mix of others		Asthma + Parkinson + cardiac diseases + pain		Cancer + diabetes + inflammatory + immune + other mental + glaucoma + HIV		N/A^d^		Hypertension		Mental diseases
Description of the clusters based on overall descriptive statistics			N/A		Average age, slightly fewer male patients, higher hospital costs and hospital stays		Average age, slightly fewer male patients, lowest deductibles, highest amount of PCGs and multimorbidity, highest health care use and costs (except for costs of medications)		Slightly older, more female patients, relatively low deductibles, high amount of PCGs (1.7) and multimorbidity (but less than cluster 0), relatively low health care use and costs		Oldest, relatively low deductibles, some complexity (more than 1 PCGs on average), very high use of doctor visits (especially generalist), many hospitalizations and high inpatient costs		Relatively old, on average 1 PCG, highest cost of medicaments, and high ambulatory costs, relatively low hospitalizations and doctor visits		Young, highest deductibles, low health care use and costs		Slightly older, more male patients, relatively low health care use and costs		Youngest, more female patients, relatively low deductibles, low health care use and costs (but higher than for hypertension group), a lot of visits to doctors

^a^PCG: pharmacy-based cost group.

^b^Ratios rounded off to one decimal place.

^c^COPD: chronic obstructive pulmonary disease.

^d^N/A: not applicable.

## Discussion

Our study shows that performing cluster analysis to explore patient multimorbidity and complexity is feasible. We demonstrated that individuals with single PCGs of mental diseases or hypertension, individuals with multiple PCGs, or individuals with a single high-cost PCG have different health care use patterns and represent different complexity groups.

Earlier studies focusing on chronic conditions identified from electronic health records evidenced the existence of systematic associations between chronic diseases, whereby chronic diseases, often from dissimilar disease categories, coappeared within a multimorbidity pattern or cluster [24-26]. Importantly, though, these studies showed that the complexity of multimorbidity patterns in terms of diseases and associated drug use increased with age, which holds true for both genders. Moreover, in line with our findings, multiple earlier studies used cluster analysis for identifying clinically homogenous multimorbidity patterns in the population, where clusters were composed of diagnosis-related groups [16,27-30]. However, these studies used measures of multimorbidity and comorbidity or clinical diagnosis data rather than PCGs from claims data. This makes direct comparison of results challenging, due to the differences in methodologies and level of diagnosis details. A recent systematic review confirmed that analytical methods used to identify patient profiles with multimorbid conditions are heterogeneous (including factor analysis, multiple correspondence analysis, hierarchical clustering, and three-step unified-clustering method), which may explain the variation in the multimorbidity patterns reported in various studies [31]. Despite those differences, the observed most prevalent clusters or groups are similar across studies and included hypertensive or metabolic diseases [28,29] and mental and behavioral diseases [16]. The greater prevalence of and similarities in metabolic and mental clusters were confirmed by a systematic review of multimorbidity patterns, whereby these clusters were identified in 9 and 10 of 14 reviewed articles, respectively [32]. One study compared multimorbidity patterns between populations of two European countries (Spain and the Netherlands) and found that, indeed, the highest similarities were observed in the cardio metabolic cluster, even though the populations are likely to differ across countries [26].

The existing literature on the use of cluster analysis to identify homogenous segments based on health care use and expenditures is limited [33-37]. Specifically, the study by Nnoaham and Cann [33] identified segments (or clusters), similar to ours, based on health care use (expressed by visits to the physicians, medications, and admissions) and complexity (expressed by long-term conditions). Other studies used cluster analysis to identify groups with high expenditures and deduced that, despite having a lot of heterogeneity, the high expenditures cluster typically exhibited fair or poor health with more medical conditions or comorbidities [34,35]. These findings confirm ours; they nevertheless need to be interpreted with caution due to differences in methodologies, age of the population, and level of details available for background individual characteristics and diagnoses. There is evidence that cluster analysis may provide more information to decision makers than a list of possible statistically significant variables or a list of individuals who are the highest users [35].

To our knowledge, this is the first study using cluster analysis to explore patients’ multimorbidity and complexity, reflected by the mix of PCGs and health care use patterns. In addition, it benefits from the richness of health care use data, a large sample size, and advanced clustering methods. However, the study has certain limitations. The first limitation stems from the process of multiple parameters configuration, which increases complexity while not allowing results validation. Thus, the cluster interpretation has to rely on metrics from the algorithms, descriptive statistics, and clinical relevance. Second, as the data were lacking clinical information, we only relied on PCGs mapping, which may give an incomplete picture of drug data [9,11,12].

Our study shows that PCG-based cluster analysis of health care use claims data allows diverting from an approach of simple comorbidity counts and can identify the population profiles with increased health care use and costs. Such results may provide insightful information for policy making, care planning, and care delivery to facilitate the transformation from procedures and guidelines focusing on a single disease toward development of integrated and better coordinated care.

## Appendix

Multimedia Appendix 1 Lists of pharmacy-based cost groups used to identify chronic diseases in insurance claims data.

## Abbreviations

DT: decision tree
HDBSCAN: hierarchical density-based spatial clustering of applications with noise
MDS: multidimensional scaling
OOB: out-of-bag
PCG: pharmacy-based cost group
RF: random forest
