# Supplementary material for: Exploring Patient Multimorbidity and Complexity Using Health Insurance Claims Data: A Cluster Analysis Approach
Source: JMIR Med Inform. 2022 Apr 4;10(4):e34274. doi: 10.2196/34274 (PMC9016510; doi:10.2196/34274)
Supplement: Multimedia Appendix 1 [file medinform_v10i4e34274_app1.docx]

**Appendix1. Table 1.** Lists of PCGs used to identify chronic diseases in insurance claims data.

| 1. *Cancer (N=1’205)* | *Cancer (kre) + Complex Cancer(krk) + Hormone sensitive tumor(kho)* |
| --- | --- |
| 1. *Inflammatory (N=387)* | *Crohn's disease/ ulcerative colitis (mcr) + Psoriasis (pso) + Rheumatism (rhe)+* *Disease of the brain or Spinal cord: multiple sclerosis (msk)* |
| 1. *Diabetes (N=4’269)* | *Diabetes type 1(dm1)+Diabetes type 2 (dm2)* |
| 1. *Hypertension related (N=20’228)* | *Diabetes with hypertension (dmh)+* *Pulmonary (arterial) hypertension(pah) + High cholesterol (hch)* |
| 1. *Immune (N=321)* | *Auto -immune disease (aik)+* *Transplants (tra)* |
| 1. *Pain (N=3’398)* | *Chronic pain (smc)+ Neuropathic pain (smn)* |
| 1. *Thyroid (N=3’355)* | *Thyroid disorder (thy)* |
| 1. *Mental (N=9’064)* | *Depression (dep)+ Bipolar disorder (bsr)+ Addiction excl. nicotine (abh) + Attention deficit hyper- activity disorder (adh)+* *Alzheimer's (alz)+ Psychosis (psy)* |
| 1. *Asthma (N=3’358)* | *Asthma (ast) + COPD/Severe asthma(cop)* |
| 1. *Parkinson (N=285)* | *Parkinson’s (par)* |
| 1. *Epilepsy (N=593)* | *Epilepsy (epi)* |
| 1. *Glaucoma (N=3’234)* | *Glaucoma (gla)* |
| 1. *HIV/AIDS (N=305)* | *HIV/AIDS (hiv)* |
| 1. *Heart disease (N=1’470)* | *Heart disease (car)* |
| 1. *Other (N=116)* | *Kidney disease (nie) + Growth disorder (was) + Cystic Fibrosis / Pancreatic Enzymes (zfp) + Diseases of the brain or Spinal cord: other (zns)* |
| 1. *No diseases (N=172’924)* | |
| 1. *Diseases (N=15’857)* | |
